# Boronated Pyrimidines and Purines as Cytotoxic, Hypolipidemic and Anti-Inflammatory Agents

**DOI:** 10.1155/MBD.1996.155

**Published:** 1996

**Authors:** Iris H. Hall, Bruce S. Burnham, Amy Elkins, Anup Sood, Walda Powell, Jeno Tomasz, Bernard F. Spielvogel

**Affiliations:** 1 Division of Medicinal Chemistry and Natural Products School of Pharmacy University of North Carolina Chapel Hill North Carolina 27599-7360 USA; 2 Boron Biologicals Inc. 620 Hutton Street Raleigh North Carolina 27606-1490 USA

## Abstract

The simple boronated bases, e.g. cytosine, adenine and guanine, containing no sugar residues retained good pharmacological activity as hypolipidemic, anti-neoplastic and anti-inflammatory agents in mice at 8 mg/kg. Their activities were generally identical to their respective nucleoside derivatives. Interestingly the boronated acyclovir derivative was a very potent hypolipidemic agent achieving better activity than clofibrate and lovastatin. The boronated adenine derivatives appeared to have the best anti-inflammatory activity in reducing local edema and analgesic effects. The agents were active against the growth of murine and human leukemias and human HeLa-S^3^ suspended uterine carcinoma. Only the boronated adenine derivatives were effective in blocking the growth of human SW480 adenocarcinoma and the KB nasopharynx.


### BORONATED PYRIMIDINES AND PURINES AS CYTOTOXIC, HYPOLIPIDEMIC AND ANTI-INFLAMMATORY AGENTS

Iris H. Hall*[1], Bruce S. Burnham[1], Amy Elkins[1], Anup Sood[2], Walda Powell[2], Jeno Tomasz[2], and Bernard F. Spielvogel[2]

[1] Division of Medicinal Chemistry and Natural Products, School of Pharmacy, University of North Carolina, Chapel Hill, North Carolina 27599-7360, USA
[2] Boron Biologicals, Inc., 620 Hutton Street, Raleigh, North Carolina 27606-1490, USA

### Abstract

The simple boronated bases, e.g. cytosine, adenine and guanine, containing no sugar residues retained good pharmacological activity as hypolipidemic, anti-neoplastic and anti-inflammatory agents in mice at 8 mg/kg. Their activities were generally identical to their respective nucleoside derivatives. Interestingly the boronated acyclovir derivative was a very potent hypolipidemic agent achieving better activity than clofibrate and lovastatin. The boronated adenine derivatives appeared to have the best anti-inflammatory activity in reducing local edema and analgesic effects. The agents were active against the growth of murine and human leukemias and human HeLa-$S^3$ suspended uterine carcinoma. Only the boronated adenine derivatives were effective in blocking the growth of human SW480 adenocarcinoma and the KB nasopharynx.

### Introduction

The anti-neoplastic activity of 2'-deoxynucleoside-cyanoboranes[1,2] has previously been reported. The original derivatives had the boron atom placed or attached on the nitrogen of guanosine, adenosine, inosine and cytidine nucleoside. The cytidine boronated nucleosides were the most potent antineoplastic/cytotoxic agents[illegible]. Further pharmacological testing showed that these boronated nucleosides also possessed hypolipidemic[3] as well as anti-inflammatory/anti-septic shock properties[4]. Subsequently, two thymidine boronated nucleosides were examined[5]. These derivatives had the boron moiety attached to the 5'-hydroxyl group of the deoxyribose sugar and possessed all three types of pharmacology activities. The derivatives were effective in reducing DNA and protein syntheses in L-1210 cells with inhibition of ribonucleoside reductase, dihydrofolate reductase, purine de novo synthesis and depending on the substituted moiety, DNA polymerase $\alpha$, and the RNA polymerase activities. d[NTP] pool levels were significantly reduced after 60 min.[illegible]. Boronated guanine and adenosine arabinoside, deoxyribose and ribose also demonstrated potent cytotoxic action[illegible]. The boronated adenosine arabinoside demonstrated very potent activity as an anti-neoplastic agent inhibiting purine synthesis and DNA synthesis but more important was the observation that it was a DNA topoisomerase II inhibitor with DNA strand scission[4]. Carborane moieties added in the 5'-hydroxyl group of the sugar residue have also demonstrated potent cytotoxic action. All of these boronated nucleosides agents have proven to be safe at their therapeutic dose in mice[7]. The current study is an investigation of the boronated bases without any sugar moieties to determine their activity as cytotoxic, hypolipidemic and as anti-inflammatory agents

### Materials and Methods

### Source of compounds

The compounds (see Fig. 1) were synthesized by a Lewis base exchange reaction described previously[8]. All radioisotopes were purchased from New England Nuclear (Boston, MA) unless otherwise indicated. Radioactivity was determined in Fisher Scintiverse scintillation fluid with correction for quenching. Substrates and cofactors were obtained from Sigma Chemical Co. (St. Louis, MO).

### Pharmacological methods

**Hypolipidemic activity:** $CF_1$ male mice (~28g) were administered compounds in 1%CMC at 8 mg/kg/day, I.P. Blood samples were obtained on days 9 and 16 between 7:30 and 8:30 a.m. Daily dosing of the agents was between 9:00 and 10:00 a.m. the serum was obtained by centrifuging the blood for 10 min. at 3500 g. The serum cholesterol levels were determined by a modification of the Liebermann-Burchard procedure[9]. Serum triglyceride were determined using a commercial kit [Boehringer Mannheim Diagnostics] .

**Cytotoxicity assays:** Compounds **1-9** were tested for cytotoxic activity by homogenizing drugs in a 1 mM solution in 0.05% Tween $80/H_2O$. These solutions were sterilized by passing them through an acrodisc filter (45$\mu$). The following cell lines were maintained by literature techniques[1,2]: murine $L_{1210}$ lymphoid leukemia, human $Tmolt_3$ acute lymphoblastic T cell leukemia, colorectal adenocarcinoma SW480, HCT-8 ileum adenocarcinoma, lung bronchogenic MB-9812, osteosarcoma TE418, KB epidermoid nasopharynx, HeLa-$S^3$ suspended cervical carcinoma, and glioma EH 118 MG. Geran et al.'s protocol[10] was used to assess the cytotoxicity of the compounds and standards in each cell line. Values for cytotoxicity were expressed as $ED_{50}$ = $\mu$g/ml, i.e. the concentration of the compound inhibiting 50% of cell growth. $ED_{50}$ values were determined by the trypan blue exclusion technique. A value of less than 4 $\mu$g/ml was required for significant activity of growth inhibition. Solid tumor cytotoxicity was determined by utilizing crystal violet/MeOH and read at 580 nm (Molecular Devices)[1,2].

**Anti-neoplastic activity:** *In vivo* antineoplastic activity was tested in the Ehrlich ascites carcinoma screen in $CF_1$ male mice[~28g]. On day zero 2 X $10^6$ Ehrlich ascites carcinoma cells were injected I.P. into the mice. Drugs were prepared on 0.05 Tween 80/water and administered I.P. on days 1-9. On day 10, the surviving animals were sacrificed and the tumor volume and astrocrit determined and the percent inhibition of tumor calculated[11].

**Anti-inflammatory screen:** Male $CF_1$ mice weighing 28-32g obtained from Jackson Lab. [Bar Harbor, MA] were administered agents at 8 mg/kg X 2 I.P. administered 3 hr and 30 min prior to administering the irritant, according to Winter's protocol[12,13]. Evaluation of the induced edema was made by injecting 2% carrageenan in 0.9% saline into the plantar region of the foot[12]. The opposite foot injected with 0.9% saline was used as a base line. The standards indomethacin (10 mg/kg) and phenylbutazone (50 mg/kg) were used to compare activity.

**Endotoxic shock: Protection against septic shock:** $CF_1$ male mice (29-31g) were administered lipopolysaccharides[LPS] Salmonella abortus equi [Lot # 69F4003] at 10 mg/kg, I.P which has an $LD_{100}$ within 48-52 hr which was consistent with literature values[14]. Drugs were administered 2 hr prior and 2 hr post-injection of the LPS and the subsequently every 24 hr for the length of the animals' lives. Deaths were recorded every 12 hr and continued for 96 hr. Indomethacin (8 mg/kg) and pentoxyfylline (50 mg/kg) were used as standards.

**Local analgesic activity:** $CF_1$ male mice (28.5-32g) were administered agents at 8 mg/kg I.P. 20 min before 0.5 mL of 0.6% acetic acid was administered I.P.[illegible]. After 5 min, the number of stretches were counted over the next 10 min. Indomethacin was used as a standard at 8 mg/kg.

**Hot plate tail flick screen:** $CF_1$ male mice (29-32g) were administered drugs at 8 mg/kg, I.P. prior to placement on a hot plate maintained at $100^{o}F$. Time elapsed prior to tail raising was measured using a digital read-out connected to the hot plate[16]. Tail flick responses of $CF_1$ mice injected with morphine were used as the standard for this assay.

### Results

The boronated purine and pyrimidine bases maintained good activity in the hypolipidemic, cytotoxicity and anti-inflammatory screens suggesting that a nucleoside form of the boronate base is not always necessary for good pharmacological activity.

Table 1. The Hypolipidemic Activity of Boronated Pyrimidine and Purine Derivatives in $CF_1$ Male Mice at 8 mg/kg/day, I.P.
Percent Control [ N = 6]

| Compound # | Serum Cholesterol | | Serum Triglyceride |
|---|---|---|---|
| | Day 9 | Day 16 | Day 16 |
| Control | 100±6 | 100±6[c] | 100±5[d] |
| **1** | 88±5 | 73±5* | 86±4 |
| **2** | 75±3* | 72±4* | 95±5 |
| **3** | 87±6 | 70±3* | 78±4* |
| **4** | 87±5 | 71±3* | 104±6 |
| **5** | 77±3* | 59±4* | 66±4* |
| **6** | 68±4* | 62±4* | 51±3* |
| **7** | 89±6 | 84±5 | 88±6 |
| **8** | 67±4* | 62±4* | 42±5* |
| **9** | 78±5* | 51±6* | 59±6* |
| Clofibrate[a] | 87±6 | 78±6* | 75±6* |
| Lovastatin[b] | 85±4 | 82±5 | 86±7 |

*$p \leq 0.001$ Student's "t" test; a = 150 mg/kg/day; b = 8 mg/kg/day, c = 125mg/dL; d = 137 mg/dL.

The cytosine derivatives [5,6,8] were better hypolipidemic agents than the simple adenine or guanine derivatives [1-4]. Compounds **5** and **7** do not contain a boron moiety yet they demonstrated significant activity. The boronated acyclovir, compound **9**, is a guanine derivative and demonstrated potent activity. Compounds **1-4** lowered serum cholesterol level on day 16, 27-30% and only compounds **1** and **4** lowered serum triglyceride levels 22% on day 16 at 8 mg/kg/day. Compounds **5-9** lowered serum cholesterol levels 38-48% and serum triglycerides 34-58%. These derivatives demonstrated activity that was superior to the standards clofibrate at 150 mg/kg/day and lovastatin at 8 mg/kg/day The methylated boronated cytosine 8 demonstrated the best overall hypolipidemic activity.

The *in vivo* anti-neoplastic activity in the Ehrlich ascites carcinoma screen showed that compounds **1-3** afforded 40%, 50%, 40% inhibition and **5**, **7** and **9** were inactive. Compounds **4**, **6** and **8** afforded 62%, 78% and 77% reduction of ascites tumor growth in mice at 8 mg/kg/day I.P.

---

Table 2 Cytotoxicity of Pyrimidine and Purine Bases

$ED_{50}$ values = μg/ml

| Cp'd # | L-1210 Leukemias | $Tmolt_3$ | HeLa-$S^3$ | Solid Uterine | KB Naso-pharynx | Skin A431 | Adenocarcinoma Colon SW-480 | Ileum HCT-8 | Lung A549 | M9812 | Osteo Bone Sarcoma | Glioma Brain |
|---|---|---|---|---|---|---|---|---|---|---|---|---|
| **1** | 1.29 | 1.98 | 0.98 | 2.34 | 2.86 | 3.18 | 1.61 | 7.98 | 4.09 | 6.02 | 7.88 | --- |
| **2** | 1.89 | 2.46 | 2.86 | 5.87 | 3.65 | 3.70 | 3.17 | 7.84 | 5.15 | 6.74 | 7.38 | --- |
| **3** | 1.59 | 1.51 | 1.38 | 5.33 | 3.82 | 6.19 | 1.08 | 7.44 | 5.07 | 6.41 | 7.81 | --- |
| **4** | 1.06 | 0.82 | 0.76 | 8.32 | 4.91 | -- | 2.53 | -- | 7.76 | 7.89 | -- | --- |
| **5** | 6.81 | 3.87 | 5.54 | 6.43 | 7.62 | -- | 7.84 | -- | 9.91 | 9.10 | 8.46 | 7.80 |
| **6** | 3.82 | 0.91 | 1.97 | 7.16 | 5.87 | -- | 7.05 | -- | 9.19 | 1.42 | 8.84 | 8.81 |
| **7** | 4.42 | 3.37 | 5.48 | 7.11 | 5.26 | -- | 6.69 | -- | 9.30 | 8.58 | 7.74 | 8.80 |
| **8** | 3.04 | 1.43 | 1.64 | 7.11 | 5.26 | -- | 6.69 | -- | 9.30 | 1.67 | 7.74 | 8.72 |
| **9** | 1.24 | 2.34 | 0.87 | 8.67 | 2.52 | -- | 10.2 | 5.96 | 8.30 | 10.23 | -- | 5.88 |
| 5FU | 1.41 | 2.14 | 2.47 | 4.11 | 1.25 | 1.12 | 3.09 | 0.61 | 3.58 | 5.64 | 3.52 | 1.28 |
| AraC | 2.76 | 2.67 | 2.13 | 4.74 | 2.84 | 2.54 | 3.42 | 0.92 | 4.69 | 6.16 | 0.86 | 1.88 |
| Hydroxyurea | 2.67 | 3.18 | 1.96 | 8.12 | 5.29 | 1.77 | 4.74 | 3.21 | 7.37 | 7.18 | 2.87 | 2.57 |
| 6MP | 2.42 | 1.62 | 2.12 | 5.61 | 11.04 | 3.61 | 3.61 | 3.42 | 4.71 | 4.29 | 9.39 | 4.46 |

---

The cytotoxicity screens showed that the adenine derivatives were all active in the L-1210 leukemia screen with $ED_{50}$ values ≤4 μg/ml.

The boronated acyclovir derivative 9 was also very active, but the cytidine derivatives without the cyano-boron moiety were not as active. The two cytidine derivatives 6 and 8 with the boron moiety were marginally active with $ED_{50}$ values ~3.0 μg/ml. All of the compounds were active against $Tmolt_3$ T cell leukemia growth. The non-boronated cytidine derivatives 5 and 7 were only marginally active. Compounds 4 and 6 afforded ED-50 values >1 μg/ml. In the HeLa-$S^3$ uterine suspended tumor screen all of the boronated compounds were active with compounds 1, 4, and 9 affording ED-50 values less than <1 μg/ml. However, only compound 1 was active against the growth of the solid HeLa uterine carcinoma. In the KB nasopharynx screen compounds 1, 2, 3, and 9 were active and in the skin epidermoid carcinoma screen compound 1 and 2 were marginally active. Colon adenocarcinoma SW480 growth was reduced by compound 1-4, only with compound 3 demonstrating the best activity resulting in an $ED_{50}$ value of 1.08 μg/ml. None of the compounds were active against HCT-8 ileum adenocarcinoma, lung A549 carcinoma, osteosarcoma and glioma growth. In the lung bronchogenic MB-9812 only compounds 6 and 8 demonstrated $ED_{50}$ values of less than 2 μg/ml.

In the anti-inflammatory screen compounds **1-3** and **6** at 8 mg/kg caused 44-51% inhibition of induced edema in mice. The remaining compounds were not active at 8 mg/kg X 2 in mice. Compound **3** was also active in the septic shock assay since it offered 66% protection at 4 or 8 mg/kg/day from death induced by LPS and was equally active as the standards petoxfylline at 50 mg/kg/day and dexamethasone 1 mg/kg/day. Compounds **1-3** inhibited the writhing reflex for local pain at 8 mg/kg and increased the tail flick assay indicative of the ability to block central pain but the compounds were not as active as morphine in this assay.

Table 3 Anti-inflammatory Activity of the Boronated Pyrimidines and Purines in $CF_1$ mice at 8 mg/kg I.P.

[N = 6]

| Compound # | Inflammation Screen | Percent Control Writhing Reflex | Tail Flick | Protection Septic Shock |
|---|---|---|---|---|
| **1** | 56±5* | 13±2* | 147 | 55 |
| **2** | 49±4* | 18±4* | 151 | 66 |
| **3** | 51±4* | 59±5* | 142 | 66 |
| **4** | 104±6 | | | |
| **5** | 105±5 | | | |
| **6** | 50±4* | | | |
| **7** | 94±6 | | | |
| **8** | 76±4* | | | |
| **9** | 72±5* | | | |
| Indomethacin @ 8mg/kg | 22±5* | | | 33 |
| Phenylbutazone@50 mg/kg | 53±4* | | | |
| Pentoxyifylline@ 50 mg/kg | 70±5* | | | 67 |
| Dexamethasone @ 1 mg/kg | | | | 67 |
| Morphine @ 1 mg/kg | | | 213 | |
| Control | 100±6 | | | |

**Discussion**

The simple bases without the ribose, deoxyribose or arabinose but having a cyanoborane moiety still maintained pharmacological activity in murine human tissue cultured cancer screens and in the *in vivo* Ehrlich screen at 8 mg/kg/day I.P. The adenine and guanine cyanoboranes demonstrated approximately the same cytotoxicity as their respective nucleoside derivatives. The adenine base where the cyanoborane moiety is on N3 maintained as good activity against the growth of those tumors as those derivatives with the cyanoborane moiety in position N1 or N7. In the cytosine series the lack of the cyanoborane moiety cause the loss of cytotoxic action and an addition methyl group in the N3 position was not effective in improving the activity. The boronated acyclovir derivative maintained good activity in the suspended tumor cell lines but was inactive against the solid tumor lines and in the *in vivo* screen.

In the anti-inflammatory screen a similar pattern was observed. The cyanoborane moiety was required for activity. A methyl substitution in the N3 position reduced the ability to block induced edema. Surprisingly, the guanine bases were able to function as analgesic agents and blocked septic or endotoxic shock induced by LPS. This was impressive in that these simple bases were equal in action to standard agents used in the clinic to protect against death from bacterial, viral, AIDS related terminal infections from septic shock.

All of the agents were effective in the hypolipidemic screens lowering serum cholesterol and in some cases serum triglyceride levels. The boronated acyclovir derivative possessed better activity than the cyanoboronated guanine. The guanine and adenine derivatives were not as active as the cytosine bases but the cyanoborane moiety was not needed by these bases to afford good activity in lowering both serum lipid levels

**References**

1. A. Sood, B.F. Spielvogel, B.R. Shaw, L.D. Carlton, B.S. Burham, E.S. Hall, and I.H. Hall, Anticancer Res. 1992, **12**, 335.
2. A. Sood, B.R. Shaw, B.F. Spielvogel, E.S. Hall, L.K. Chi, and I.H. Hall, Pharazie 1992, **47**, 833.
3. I.H. Hall, B.S. Burham, K.G. Rajendram, S.Y. Chen, A. Sood, B.F. Spielvogel, and B.R. Shaw, Biomed. Pharmacotherp 1993, **47**, 79.
4. K.G. Rajendram, B.S. Burham, S.Y. Chen, A. Sood, B.F. Spielvogel, B.R. Shaw, and I.H. Hall, J. Pharm. Sci. 1994, **83**, 1391.
5. I.H. Hall, E.S. Hall, L.K. Chi, B.R. Shaw, A. Sood, and B.F. Spielvogel, Anticancer Res. 1992, **12**, 1091.
6. A. Sood, B.F. Spielvogel, W.J. Powell, Ken F. Bastow, M.C. Miller, III, and I.H. Hall, Anticancer Res. 1994, **14**, 1483.
7. I.H. Hall, B.D. Burham, K.G. Rajendram, J.J. Chang, A. Sood, and B.F. Spielvogel, Metal-Based Drugs 1994, **1**, 19.
8. B.F. Spielvogel, A. Sood, J. Tomasz. B.R. Shaw, B.S. Burnham, I.H. Hall, Boron U.S.A. III, Abstract 54, Pullman WA, July 8-11, 1992.
9. A.T. Ness, J.V. Pastewka, A.C. Peacock, Clin. Chem. Acta 1964,**10** 229.
10. R.J. Geran, N.H. Greenburg, M.M. MacDonald, A.M. Schumacher, B. J. Abbott, Cancer Chemo Rep 1972; **3**, 9.
11. C. Piantadosi, C.S. Kim, and J.L. Irvin, J. Pharm. Sci. 1969, **58**, 821.
12. C.A. Winter, E. Risley and G. Nuss, Proc. Soc. Exp. Biol. Med., 1962, **111**, 544.
13. A.P. Roszkowski, W.h. Rooks, A. Tomomlonis and L.M. Miller, J. Pharm. Exp. Ther. 1971, **179**, 114 .
14. L. Noronha-Blob, V.C. Lowe, M. Weitzberg and R.M. Burch, Eur. J. Pharm. 1991, **199**, 387.
15. L.C. Hendershot and J. Forsaith, J. Pharmacol. Exp. Ther. 1959, **125**, 237.
16. I.H. Hall, J.E. Hall, Jr., M. Mohseni and Z. Sajadi, J. Pharm. Sci. 1980, **69**, 1451.

**Received: April 19, 1996 - Accepted: May, 1996 -**

**Received in revised camera-ready format: May 27, 1996**

